# Efficacy of a yeast postbiotic on cold/flu symptoms in healthy children: A randomized-controlled trial

**DOI:** 10.1038/s41390-024-03331-z

**Published:** 2024-06-28

**Authors:** Ruma G. Singh, Vicenta Garcia-Campayo, Justin B. Green, Neil Paton, Julissa D. Saunders, Huda Al-Wahsh, David C. Crowley, Erin D. Lewis, Malkanthi Evans, Marc Moulin

**Affiliations:** 1 KGK Science Inc., London, ON Canada; 2 Cargill Limited c/o Cargill Inc., Wayzata, MN USA; 3 Department of Biochemistry, Western University, London, ON Canada

## Abstract

**Background:**

Children attending school/daycare are at high risk of acute respiratory tract infections. EpiCor^TM^ postbiotic, derived from yeast fermentate, has been demonstrated to improve immune function in adults, reducing the incidence of cold/flu-like or allergy symptoms. As such, studies are warranted in children as available pharmaceutical options have unwanted side effects.

**Methods:**

Two-hundred and fifty-six children aged 4–12 years attending school/daycare were randomized to either EpiCor or Placebo for 84 days during the 2022–2023 flu season in Ontario, Canada. The Canadian Acute Respiratory Illness and Flu Scale (CARIFS) and study diary assessed the incidence and severity of cold/flu symptoms and the use of cold/flu medications. Adverse events were recorded.

**Results:**

Total CARIFS severity scores, ‘sore throat’ and ‘muscle aches or pains’ symptom scores in the EpiCor group were significantly lower compared to Placebo during incidences of cold/flu (*P* ≤ 0.05). Participants taking Placebo were 1.73 times more likely to use cold/flu medication compared to those receiving EpiCor (*P* = 0.04). The incidence of cold/flu symptoms was not significantly different between groups. EpiCor was found to be safe and well-tolerated.

**Conclusions:**

EpiCor supplementation resulted in significantly lower cold/flu symptom severity and less cold/flu medication usage than Placebo demonstrating a beneficial effect on immune function in children.

**Impact:**

- Children are at high risk of acquiring cold/flu infections and safe and efficacious mitigating regimens are lacking.
- Children supplemented daily with 500 mg EpiCor^TM^ postbiotic derived from yeast fermentate had significantly lower overall cold/flu symptom severity, and severity of sore throat and muscle aches or pains over the 84-day supplementation period.
- EpiCor supplementation resulted in decreased use of traditional cold/flu medication.
- Daily supplementation with 500 mg of EpiCor for 84 days was safe and well tolerated by healthy children aged 4–12 years attending school or daycare.

## Introduction

Acute upper respiratory tract infections (URTIs) are the most frequently reported health problems in children.^1^ Despite the common cold/flu being a self-limiting viral infection, visits to physicians, treatments, absenteeism from school or daycare as well as absenteeism of caregivers from work, contribute to a substantial economic burden. On average, children can have 7–10 cold/flu incidences per year, with higher incidences for children attending daycare or school.^2,3^ Cold/flu medications used to alleviate symptoms such as codeine, hydrocodone, and decongestants are not recommended for children and there is little evidence that over-the-counter (OTC) medications are effective for this age group.^4^ Further, overuse and misuse of antibiotics in pediatric populations is associated with growing antibiotic resistance and side effects, such as rash, diarrhea, and allergic reactions.^5,6^ Therefore, additional safe and efficacious solutions are needed to manage cold/flu and to decrease their incidence, severity, and duration in children.

Dietary supplements such as echinacea, zinc, elderberry, and β-glucan have been investigated for their effects on reducing or treating cold symptoms. However, there are inconsistencies in the findings or insufficient clinical evidence for these supplements demonstrating a beneficial effect in children.^7–10^ A recent systematic review and meta-analysis reported that probiotics may reduce the incidence of acute URTIs in children, however, effects on the duration or severity of cold symptoms were not reported.^11^ Overall, not much is known about the safety of these supplements in children with only a limited number of published studies in the pediatric population.

Furthermore, accumulating evidence suggests that the viability of microorganisms included in probiotics are not essential for eliciting beneficial effects on human health.^12,13^ Administration of compounds composed of non-viable micro-organisms often with their metabolites,^14^ commonly known as postbiotics, offers safety advantages over probiotics, as there is no risk of intestinal translocation, bacterial resistance, or worsening of local inflammation.^15^ Given these advantages, postbiotic supplementation may be a promising alternative for reducing the incidence of infectious diseases in pediatric populations.^15,16^ EpiCor postbiotic has been demonstrated to improve immune function in healthy adults, lessening the impact or incidence of cold/flu-like or allergy symptoms.^17–19^ However, the effects of postbiotic supplementation on immune function in the developing immune systems of children are not well understood. The objective of this study was to investigate the efficacy and safety of 84-day supplementation of EpiCor on the incidence of cold/flu symptoms among children aged 4–12 years.

## Methods

### Study design and population

**Fig. 1:**
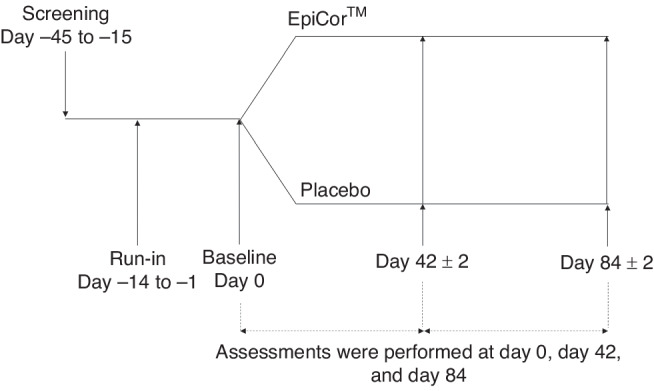
Study design. Participants underwent a 14-day run-in period prior to baseline (Day 0). At Day 0, eligible participants were randomized equally to one of the two study arms in a double-blinded manner. In-clinic study assessments were conducted at Days 0, 42, 84.

The study consisted of a 14-day run-in period followed by an 84-day supplementation period in which participants were randomized equally (1:1 ratio) to receive either EpiCor or Placebo (Fig. 1). Study assessments were performed at day 0 (baseline), 42, and 84. The study timeline coincided with the flu season in Ontario, Canada, which typically occurs between late November and March.^21,22^ Participants were males and females between 4-12 years of age, enrolled in and attending school or daycare, undertook together with their guardian to maintain their current lifestyle habits for the duration of the study, and had a care provider who could reliably bring them to all study visits. Participants were healthy as determined by medical history and a review of their health status by the Medical Director (MD).

Individuals were excluded if they had an allergy, sensitivity, or intolerance to investigational product (IP) active or inactive ingredients, had an immune dysfunction and/or were taking immunosuppressive medication, used concomitant prescribed (e.g., cyclosporine, methotrexate, azathioprine) or over-the-counter supplements (e.g., products containing components of *Saccharomyces cerevisiae* [e.g., β-glucan]), had severe environmental allergies requiring medication or need for allergy shots, asthma, a history or presence of a clinically relevant cardiac, renal, hepatic, endocrine, pulmonary, biliary, gastrointestinal or pancreatic disorder, or any other condition that may have adversely affected ability to complete the study or its measures or which may have posed significant risk to the participant, as assessed by the MD. Further, sibling participants who lived in the same household could not be enrolled at the same time.

### Investigational products and Placebo

Given high adoption among children and improved compliance, a gummy format was utilized as the delivery method for this trial. Each gummy of EpiCor contained 250 mg of dried yeast fermentate and excipients identical to Placebo. The Placebo contained tapioca syrup, cane sugar, purified water, pectin, citric acid, natural flavours, sodium citrate, and natural colour. The gummies were manufactured by Viva 5 (St. Petersburg, FL).

Participants were required to take two gummies per day (IP or Placebo) in the morning, with or without food, for 84 days. If a participant missed a morning dose, they were instructed to take the missed dose that day as soon as they remembered. Participants were advised not to exceed two gummy supplements daily.

### Randomization and blinding

Participants were assigned a randomization number derived from the randomization list (www.randomization.com) by a blinded investigator. Investigators, study personnel, and participants/caregivers were blinded to the products. EpiCor and the Placebo were similar in appearance to ensure allocation concealment. The study products were sealed in identical bottles and labelled by personnel not involved in study assessments per the requirements of the International Council for Harmonization of Technical Requirements for Pharmaceuticals for Human Use (ICH) Guideline for Good Clinical Practice (GCP) and applicable local regulatory guidelines.

### Study outcomes

The primary outcome was the difference in the incidence of cold/flu symptoms between EpiCor and Placebo over the 84-days supplementation period, as measured by the Canadian Acute Respiratory Illness and Flu Scale (CARIFS).

Secondary outcomes included the difference between EpiCor and Placebo in cold/flu incidence over the 42-day supplementation period, the difference between EpiCor and Placebo in cold/flu symptom duration and severity, the number of days prior to first cold/flu symptoms, the number of missed school or daycare days due to cold/flu symptoms, number of well days related to the absence of cold/flu symptoms, the proportion of participants with no cold/flu symptoms, and use of prescription and non-prescription cold/flu medications over 42 and 84 days of supplementation.

Other secondary outcomes included the difference between the EpiCor and Placebo groups in saliva secretory immunoglobulin A (sIgA) concentration and in quality of life following 42 and 84 days of supplementation. Safety was assessed by adverse events (AEs).

### Study assessments

#### CARIFS and study diary

The CARIFS is a validated tool used to assess cold/flu symptom severity in children.^23–25^ It includes 18 items answered on a four-point scale (0 = no problem, 1 = minor problem, 2 = moderate problem, 3 = major problem) across three domains: symptoms, function, and parental impact.^23^ The study diary included reporting of missed school or daycare days, any other changes in school or daycare, use of cold/flu medications (including antibiotics for cold/flu and antiviral pharmaceuticals), product use (time of day), concomitant therapies, health changes, and AEs. The CARIFS was included as a part of the study diary. The following study outcomes were assessed using CARIFS and the study diary.

Incidence of cold/flu symptoms was defined as two or more consecutive days with scores above zero (no problem) in at least two symptoms (headache, sore throat, muscle aches or pain, fever, cough, nasal congestion or runny nose, and vomiting; items 10–16) listed on the CARIFS. This definition has been previously used in a population of healthy children between 1–6 years of age attending daycare.^25^

Symptom duration was defined as the length of time from the onset of an incidence until the last day with symptoms, followed by two consecutive days with a CARIFS symptom score of zero.

Symptom severity was calculated using two methods: first, the mean total CARIFS score and mean severity scores of individual items on the CARIFS per day per incidence of cold/flu symptoms, and second, the area under the curve (AUC) of daily symptom scores (CARIFS items 10–16). The number of days prior to the first cold/flu symptoms was calculated by counting the number of days prior to first incidence of cold/flu (as defined above).

The number of missed school or daycare days due to cold/flu symptoms was calculated by counting days where caregivers of participants reported a ‘yes’ for missed school or daycare days in the study diary and reported scores > 0 for two or more CARIFS symptoms.

The number of well days was determined by counting the number of days with a score of zero for items 10–16 on CARIFS.

The proportion of children with no cold/flu symptoms was calculated by including all participants reporting a CARIFS symptom score of zero for the duration of the supplementation period.

#### Quality of life

Quality of life was assessed by the KINDL^R^ questionnaire, a validated measure of quality of life in children 3–17 years of age.^26,27^ Caregivers completed one of two parental versions of the KINDL^R^ questionnaire depending on the age of their child: the Kiddy KINDL^R^ (3–6 years old) and standard KINDL^R^ (7–17 years olds). Each version contained the same 24 questions answered on a five-point scale by the caregiver (1 = never, 2 = seldom, 3 = sometimes, 4 = often, 5 = all the time), with minor differences in language to account for the age range of respondents.

#### Saliva secretory immunoglobulin A (sIgA) analysis

At each study visit, parents/caregivers reviewed passive drool collection instructions with study personnel and were provided a copy of instructions to take home to collect saliva samples from participants. An instructional video link was also provided to participants for further guidance. Participants collected saliva samples at home in cryogenic vials, in the morning between 0700 h and 0900 h prior to each clinic visit. Participants were instructed to not eat or drink and avoid teeth brushing 60 and 45 minutes prior to collection, respectively. Two samples with a minimum volume of 1 mL of saliva per vial were collected to ensure adequate volume for analysis of sIgA. If there was inadequate volume in one vial, both samples were sent for analysis. The collected saliva samples for sIgA were transported to Salimetrics, LLC (Carlsbad, CA) for sIgA concentration analysis. sIgA concentration was analyzed using the Salimetrics^®^ SIgA Indirect Enzyme Immunoassay Kit (Cat. No. 1-1602; Salimetrics, PA).

#### Safety

Safety was assessed by AEs which were reported in the study diary. The severity of an AE was classified as ‘mild’, ‘moderate’, ‘severe’, or ‘life-threatening’, and the degree of relationship between the study product and an AE was categorized as ‘not related’, ‘unlikely’, ‘possibly’, ‘probably’, and ‘definitely’, as determined by the MD.

### Recruitment and screening procedures

Potential participants were recruited from a database of existing study participants, social media, and local community outreach initiatives including sporting events and festivals. Interested caregivers completed an online screening questionnaire and based on those responses were invited to complete an in-person screening visit. An informed consent form (ICF) was provided to potential volunteers and their caregivers to review and ask questions. Upon signing the ICF, assessments at the screening visit included a review of medical history, concomitant therapies, health status, and eligibility criteria. Vital signs (heart rate and blood pressure), weight, and height were taken and all relevant study materials including saliva collection kit, study diaries and CARIFS questionnaire with corresponding instructions were provided to the participants.

### Study procedures

Participant caregivers completed the study diary daily, including the CARIFS, starting on day -14 (the first day of the run-in period) through to the end of the study period. Participants reported to the study clinic at baseline, Day 42, and Day 84 with their saliva samples, during which they completed the KINDL^R^ questionnaire, and returned their study diary and unused IP. Compliance was calculated by determining the number of dosage units taken divided by the number of dosage units expected to have been taken multiplied by 100. Urine pregnancy tests for females of child-bearing potential were completed at screening and baseline and clinical assessments for anthropometrics and vitals were conducted at each clinic visit.

### Statistical analyses

A total of 256 subjects (128 per group) was previously estimated to be large enough to detect a difference in mean change in the incidence of common cold/flu-like symptoms of 0.19 between the IP and Placebo groups from baseline to end-of-study, with 90% power, at a 5% significance level, and a 20% dropout rate.^18^

Summary statistics including mean, median, standard deviation, first quartile, and third quartile are presented for continuous outcomes for each group with frequencies and proportions presented for categorical outcomes. The primary outcome and the binary secondary outcomes were assessed using Z test, Fishers exact test, and logistic regression. Continuous secondary outcomes were assessed using a two-sample *t*-test Wilcoxon’s rank sum test or linear mixed models. Count outcomes were assessed using a two-sample *t*-test or Wilcoxon’s rank sum test and negative binomial models. All statistical analyses were performed using the R Statistical Software Package Version 4.2.1. *P*-values ≤ 0.05 were considered statistically significant.

The intention-to-treat (ITT) population consisted of all participants who received either product and on whom any post-randomization efficacy information was available. Analyses are reported for the Per Protocol (PP) population consisting of all participants who consumed at least 80% of EpiCor or Placebo doses, did not have any major protocol violations related to the primary outcome, and completed all study visits and procedures connected with measurement of the primary variable. In the analysis performed on the PP population, crude models, consisting of only one independent variable (EpiCor vs. Placebo), were used. Responder subgroup analysis based on sIgA concentrations was conducted for participants in the PP population. Responders were defined as participants who experienced increases in sIgA from baseline at day 84, and non-responders were defined as those who experienced decreases in sIgA from baseline at day 84.

## Results

### Study population

**Fig. 2:**
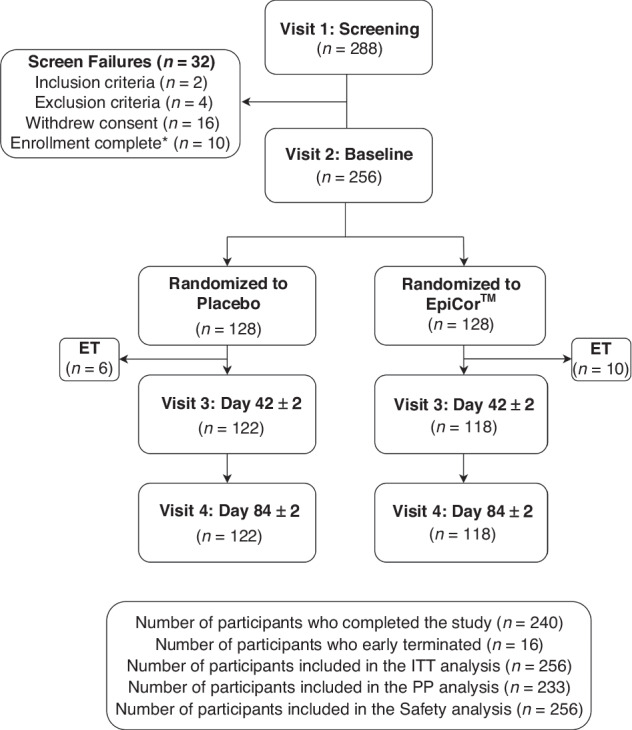
Disposition of study participants. *Includes participants who screen-failed during the screening period as enrollment of 256 participants was completed prior to their randomization.

A total of 288 volunteers were screened and consented to participate in the study with 256 participants enrolled in the current study in the ITT population (Fig. 2). Twenty-three participants were excluded from the PP population due to early termination (*n* = 16), <80% IP compliance (*n* = 3), and out of window study visit (*n* = 4) with 15 and 8 participants excluded from the EpiCor and Placebo groups, respectively. Therefore, the PP population consisted of 233 participants including 132 males and 101 females, with an average age of 8.76 ± 2.46 years. There were no significant differences in demographics, anthropometrics, flu or COVID-19 vaccination status between groups (Table 1). Study product compliance was 97.63 ± 4.35% for those supplemented with EpiCor and 98.43 ± 3.08% for those on Placebo (*P* = 0.29). Similarly, for the full study population (ITT), compliance was 93.08 ± 16.89% for those supplemented with EpiCor and 95.51 ± 14.44% for those on Placebo (*P* = 0.12).

**Table 1:** Characteristics of study participants (*n* = 233).

Variable	EpiCor (*n* = 113)	Placebo (*n* = 120)	*P*-value between EpiCor and Placebo Groups
Age (years)			
Mean ± SD	8.63 ± 2.46	8.89 ± 2.46	0.39^a^
Median (Q1 to Q3)	9.00 (6.00 to 11.00)	9.00 (7.00 to 11.00)	
Sex (***n*, %)**			
Female	48 (42.48%)	53 (44.17%)	0.90
Male	65 (57.52%)	67 (55.83%)	
Weight (kg)			
Mean ± SD	33.97 ± 12.79	35.97 ± 14.76	0.40^a^
Median (Q1 to Q3)	32.20 (23.70 to 40.70)	32.85 (23.93 to 45.65)	
Height (cm)			
Mean ± SD	136.64 ± 15.81	138.28 ± 17.53	0.46^a^
Median (Q1 to Q3)	137.90 (124.50 to 149.00)	138.35 (124.38 to 151.93)	
Race (***n*, %)**			
African American or Black	5 (4.42%)	3 (2.50%)	0.62
Central American	1 (0.88%)	2 (1.67%)	
East Asian	1 (0.88%)	1 (0.83%)	
Eastern European White	12 (10.62%)	7 (5.83%)	
Hispanic or Latino	1 (0.88%)	6 (5.00%)	
Middle Eastern	4 (3.54%)	3 (2.50%)	
Mixed	9 (7.96%)	14 (11.67%)	
Native or First Nations	0 (0.00%)	1 (0.83%)	
Other	4 (3.54%)	4 (3.33%)	
South American	1 (0.88%)	2 (1.67%)	
South Asian	1 (0.88%)	3 (2.50%)	
South East Asian	1 (0.88%)	0 (0.00%)	
Western European White	73 (64.60%)	74 (61.67%)	
Ethnicity (***n*, %)**			
Hispanic or Latino	4 (3.54%)	12 (10.00%)	0.07
Not Hispanic or Latino	109 (96.46%)	108 (90.00%)	
COVID-19 vaccine (*n*, %)*			
Yes	22 (19.47%)	24 (20.00%)	1.000
No	91 (80.53%)	96 (80.00%)	
Flu vaccine (***n*, %)**			
Yes	9 (7.96%)	4 (3.33%)	0.157
No	104 (92.04%)	116 (96.67%)	

*n* number of participants, *SD* standard deviation, *Q1* first quartile, *Q3* third quartile.

*Participants with ≥2 doses at baseline.

*P*-values for categorical variables were calculated using Fisher’s exact test.

*P*-values for continuous variables were calculated using *t*-test.

^a^*P*-values for continuous variables were calculated using Wilcoxon-test.

### Incidence of cold/flu symptoms

A total of 64.60% of participants in the EpiCor group and 74.17% of participants in the Placebo group had at least one incidence of cold/flu symptoms over the 84-day supplementation period (*P* = 0.15) (Table 2). Over the first 42 days, 47.79% of the EpiCor group and 54.17% of the Placebo group had at least one incidence of cold/flu symptoms (*P* = 0.40) (Table 3).

**Table 2:** Proportion of participants with the incidence of cold or flu symptoms over 84 days of supplementation (*n* = 233).

	EpiCor (*n* = 113)	Placebo (*n* = 120)	*P*-value^a^ Between EpiCor and Placebo Groups	*P*-value^b^ Between EpiCor and Placebo Groups
			Difference [95% CI]	Odds ratio [95% CI]
**No cold or flu incidence**	40 (35.40%)	31 (25.83%)	0.15	0.11
			0.10 [−0.03 to 0.22]	0.64 [0.36 to 1.11]
**≥1 incidence**	73 (64.60%)	89 (74.17%)	0.15	0.11
			−0.10 [−0.22 to 0.03]	1.57 [0.90 to 2.77]
**≥2 incidences**	36 (31.86%)	51 (42.50%)	0.12	0.09
			−0.11 [−0.24 to 0.03]	1.58 [0.93 to 2.72]
**≥3 incidences**	22 (19.47%)	21 (17.50%)	0.83	0.70
			0.02 [−0.09 to 0.13]	0.88 [0.45 to 1.70]
**≥4 incidences**	10 (8.85%)	9 (7.50%)	0.89	0.71
			0.01 [−0.07 to 0.09]	0.84 [0.32 to 2.15]
**≥5 incidences**	2 (1.77%)	1 (0.83%)	NA	NA
			NA	NA

*NA* not applicable because of the small number of incidences, *CI* confidence interval.

^a^*P* values and 95% CI were calculated using two proportion z-test.

^b^*P* values and 95% CI were calculated using logistic model.

**Table 3:** Proportion of participants with incidence of cold or flu symptoms over 42 days of supplementation (*n* = 233).

	EpiCor (*n* = 113)	Placebo (*n* = 120)	*P*-value^a^ Between EpiCor and Placebo Groups	*P*-value^b^ Between EpiCor and Placebo Groups
			Difference [95% CI]	Odds ratio [95% CI]
No cold or flu incidence	59 (52.21%)	55 (45.83%)	0.40	0.33
			0.06 [−0.07 to 0.20]	0.77 [0.46 to 1.30]
≥1 incidence	54 (47.79%)	65 (54.17%)	0.40	0.33
			−0.06 [−0.20 to 0.07]	1.29 [0.77 to 2.17]
≥2 incidences	20 (17.70%)	18 (15.00%)	0.70	0.58
			0.03 [−0.08 to 0.13]	0.82 [0.41 to 1.65]
≥3 incidences	3 (2.65%)	3 (2.50%)	NA	NA
			NA	NA

*NA* not applicable because of the small number of incidences, *CI* confidence interval.

^a^*P* values and 95% CI were calculated using two proportion z-test.

^b^*P* values and 95% CI were calculated using logistic model.

### Severity of cold/flu symptoms

**Fig. 3:**
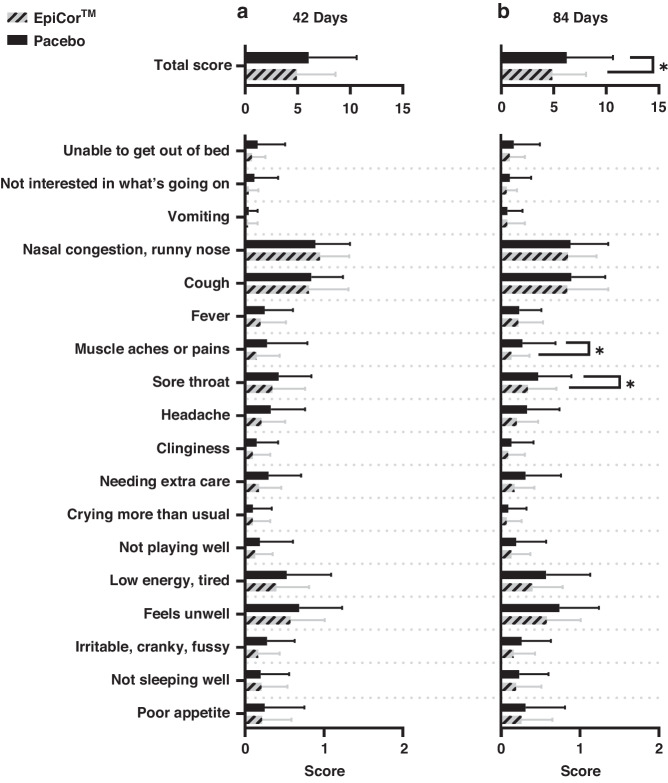
Severity of cold/flu symptoms as assessed by the Canadian Acute Respiratory Illness and Flu Scale (CARIFS). Severity of cold/flu symptoms over (**a**) 42 days and (**b**) 84 days of supplementation using the CARIFS total score and scores of individual items. *Indicates a significant difference between the EpiCor and Placebo groups; data presented as mean ± standard deviation.

The EpiCor group had a significantly lower total CARIFS severity score of 4.88 ± 3.20 compared to 6.23 ± 4.40 in the Placebo group during incidences of cold/flu over supplementation period (*P* = 0.02). Over 84 days, the mean severity scores for 17 of the 18 CARIFS items were numerically lower for the EpiCor group versus the Placebo group, with only “sore throat’ (0.34 ± 0.36 and 0.13 ± 0.23, for the EpiCor group and Placebo group, respectively (*P* = 0.04)) and ‘muscle aches or pains’ (0.47 ± 0.42 and 0.27 ± 0.42 (*P* = 0.05)) showing statistically significant differences (Fig. 3). There were no significant between-group differences in AUC for total CARIFS daily symptoms scores over 42 (EpiCor vs. Placebo, 22.56 ± 33.23 vs. 21.04 ± 23.06, *P* = 0.43) or 84 days (41.86 ± 52.96 vs. 39.57 ± 32.85, *P* = 0.28).

### Use of cold/flu medication over the 84-day supplementation period

**Fig. 4:**
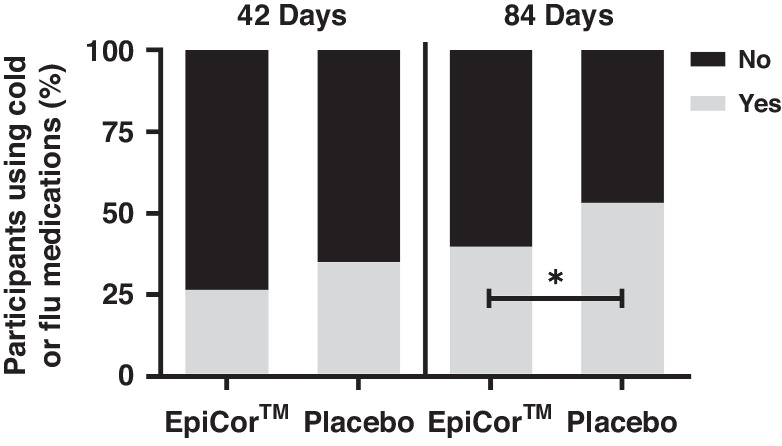
Participants using cold/flu medications during the study period. *Indicates a significant difference in the odds of using cold/flu medication between the EpiCor and Placebo groups.

Participants taking Placebo were 1.73 [1.03 to 2.92] (Odds ratio [95% CI]) times more likely to use cold/flu medication compared to those supplemented with EpiCor (*P* = 0.04) over the 84-day study period. These results are supported by a greater proportion of participants in the Placebo group (53.33%) than the EpiCor group (39.82%) that used cold/flu medication during the 84 days of supplementation (*P* = 0.05) (Fig. 4). There were no significant between-group differences in the proportion of participants using cold/flu medication over the first 42 days of supplementation. Specific use of antibiotics and inhalers inclusive of cold/flu illness is shown in Supplementary Table 1. There was no significant difference in the use of antibiotics during the study period between EpiCor and Placebo groups (*P* = 0.63). Related to medication use, there were 16 participants in the EpiCor group and 10 participants in the placebo group who visited the doctor/nurse practitioner due to cold/flu symptoms during the study.

### Duration of cold/flu symptoms

Amongst participants with confirmed cold/flu incidence, the mean duration of cold/flu symptoms in EpiCor and Placebo groups were 8.27 ± 7.35 days and 7.42 ± 7.84 days (*P* = 0.14), respectively, over 84 days. The number of days prior to first cold/flu symptoms in EpiCor and Placebo groups during 84 days of supplementation were 23.19 ± 23.97 days and 27.31 ± 24.06 days, respectively (*P* = 0.13). There were no significant between-group differences in the duration of cold/flu symptoms over the first 42 days of supplementation.

### Number of well days and number of missed school or daycare days

During 84 days of supplementation, the number of well days related to the absence of cold/flu symptoms was 65.55 ± 17.67 days for participants supplemented with EpiCor compared to 66.89 ± 14.68 days for those taking Placebo (*P* = 0.83). There were no significant differences in the proportion of participants with no cold/flu symptoms over 42 (12.39% in EpiCor vs. 12.50% in Placebo) and 84 days (5.31% in EpiCor vs. 4.17% in Placebo) of supplementation (*P* > 0.9). The number of missed school or daycare days due to cold/flu were 2.06 ± 2.92 days in the EpiCor group and 2.14 ± 2.27 days in the Placebo group (*P* = 0.22). There were no significant between-group differences in the number of well days or missed school/daycare days over the first 42 days of supplementation.

### Quality of life

There were no significant differences in total KINDL^R^ score or scores for individual domains of ‘family’, ‘friends’, and ‘functioning’ after 42 or 84 days of supplementation. At day 84, participants on EpiCor had significantly greater scores for ‘psychological well-being’ (89.10 ± 8.83) compared to those on Placebo (86.46 ± 10.45) (*P* = 0.05). The Placebo group had a significant decrease in their ‘physical well-being’ (-3.18 ± 18.58, *P* = 0.04) and an improvement in ‘self-worth’ (3.23 ± 13.61, *P* = 0.01)) from baseline at day 84.

### Saliva secretory immunoglobulin A (sIgA) concentration

**Fig. 5:**
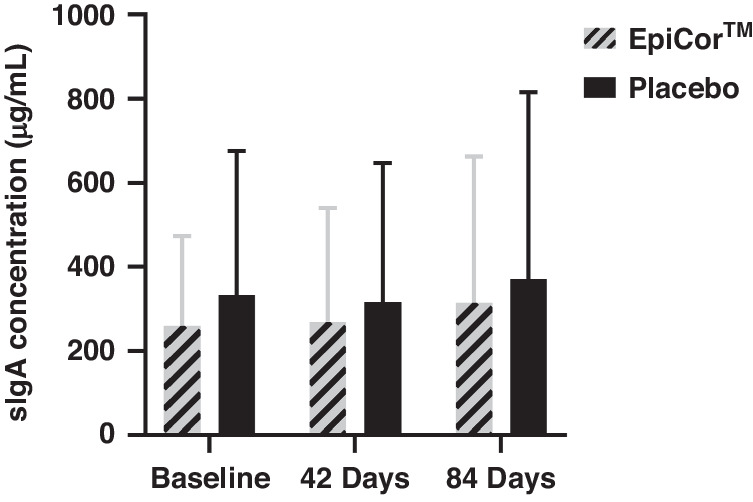
Saliva secretory immunoglobulin A concentrations. Saliva secretory immunoglobulin A concentrations in EpiCor and Placebo groups at 42 and 84 days of supplementation; data presented as mean ± standard deviation.

sIgA concentration increased by 21.5% from baseline at day 84 in participants supplemented with EpiCor (*P* = 0.10) compared to an increase of 11.7% in those on Placebo (*P* = 0.24). There were no significant between-group differences in sIgA concentration after 42 or 84 days of supplementation (Fig. 5).

### Responder population subgroup analyses

The EpiCor and Placebo groups had 59 and 68 responders respectively. There were no significant differences between the EpiCor and Placebo groups in the incidence of cold/flu over 84 days of supplementation (*P* = 0.09). There were significant improvements in sIgA concentration from baseline at days 42 and 84 in the EpiCor group with increases of 62.6% and 136.8%, respectively (*P* ≤ 0.02), and a significant sIgA increase in the Placebo group at day 84 only (89.5%; *P* < 0.01). There were no other significant differences in the responder population.

### Safety

Supplementation with EpiCor for 84 days was safe and well tolerated in the studied population. A total of 89 post-emergent AEs were reported by 67 unique participants, with 45 AEs in the EpiCor group and 44 AEs in the Placebo group. Of these AEs, all were either “unlikely” related to the study product (3 in the EpiCor group and 1 in Placebo) or “not related” (41 in the EpiCor group and 43 in the Placebo group), except for one instance of diarrhea classified as “possibly” related to the study product in the EpiCor group. However, this AE was of moderate intensity and the participant recovered three days after onset. All AEs resolved by the end of the study period or upon subsequent follow-up except in one instance of T-cell lymphoma, diagnosed after a baseline visit (participant dropped out of the study) and one instance of hair loss, both of which were deemed not related to the study product by the MD and therefore did not require follow-up.

## Discussion

This double-blind, randomized, placebo-controlled, parallel study investigated the efficacy and safety of EpiCor postbiotic on cold/flu symptoms in healthy children aged 4–12 years over the November 2022 – March 2023 flu season in Ontario, Canada. A dose of 500 mg/day EpiCor was chosen based on the safety and efficacy established in a previous adult study showing a high rate of compliance to study product.^19^ Compliance to EpiCor was greater than 90%, demonstrating acceptance from younger individuals who may be more particular with the food products they consume. Daily oral supplementation with 500 mg EpiCor, in a gummy format, for 84 days did not significantly reduce the incidence of cold/flu symptoms but resulted in significantly lower overall cold/flu severity compared to Placebo, demonstrating a beneficial effect of EpiCor on immune function in children. This was supported by lower severity of individual symptoms of ‘sore throat’ and ‘muscle aches or pain’ compared to those taking Placebo. Notably, the positive findings in this study were demonstrated in a large sample size of 233 children attending school or daycare.

The immune response to a viral infection is the main factor in generating cold/flu symptoms, which consists of production of proinflammatory cytokines and mediators.^28^ Cold symptoms can be categorized as respiratory and systemic symptoms, with sore throat being one of the first respiratory symptoms to develop after a rhinovirus infection.^28^ Sore throat is caused by localized stimulation of sensory nerve endings by prostaglandins and bradykinin in the respiratory tract^29,30^ and may contribute to painful swallowing, decreased appetite, and dehydration. Mitigating the severity of sore throat in children may be beneficial as throat irritation may progress into throat pain associated with nasopharyngitis or tonsilitis.^31^ Further, proinflammatory cytokine stimulation of prostaglandins and their effect on sensory nerves in skeletal muscles results in muscle aches during cold/flu infection,^32^ negatively impacting an individual’s activity. EpiCor contains bioactive compounds, such as vitamins and minerals, cell wall components including beta glucans known as immune modulators, and polyphenols. Previous pre-clinical and clinical studies in adults demonstrated anti-inflammatory and anti-oxidative properties of EpiCor which facilitates resolution of inflammatory responses, improves gastrointestinal health, and boosts mucosal immunity.^33–36^ The findings from the current study demonstrate that EpiCor supplementation reduces the burden of symptoms that may otherwise impact the activity level and daily routine of children. Further, the effect of EpiCor on symptom severity may have been mediated by sIgA, a mucosal antibody that serves as the first line of defense to counter infections.^37^ There is evidence suggesting that anti-inflammatory effects of sIgA following an infection plays an important role in maintaining mucosal homeostasis by limiting exacerbation of inflammatory responses.^38,39^ In this study, sIgA concentration increased by 21.5% from baseline at day 84 in participants supplemented with EpiCor compared to an increase of only 11.7% in Placebo. Furthermore, subgroup analysis of the responder population showed that there were significant improvements in sIgA concentration throughout the supplementation period in the EpiCor group, with increases of 62.6% and 136.8% from baseline at days 42 and 84, respectively (vs. 89.5% increase at day 84 only in Placebo). Taken together, the findings suggest that reduced cold/flu severity in participants supplemented with EpiCor may have been associated with greater increases in sIgA concentration. To further elucidate this association and mechanism of action, future research could include correlation analysis between sIgA concentrations and timing of cold/flu symptoms. It is also possible that increases in sIgA concentrations may have been related to other immunological changes over the study period, which warrants further investigation.

Use of prescription and non-prescription cold/flu medication likely correlates with greater symptom severity as parents are more inclined to give their child these types of rescue medications if their child’s symptoms are problematic, or severe.^40^ Over-the-counter medications consumed by participants in this study included acetaminophen, ibuprofen, antihistamines, and dextromethorphan. These are the most commonly consumed medications for cough and cold in children.^41^ However, there is evidence that some of these medications are not effective in the treatment of URTIs experienced during childhood.^42^ Moreover, side-effects associated with these medications may be problematic. In an open-label study of 424 children with fever and/or pain, consumption of ibuprofen and acetaminophen was associated with abdominal pain, hyperkinesia, insomnia, stupor, twitch, atelectasis, sweat, and bodily pain.^43^ Another randomized clinical trial involving children aged 1-12 years old with URTIs reported that participants experienced abdominal pain, nausea, vomiting, drowsiness, and irritability following consumption of dextromethorphan.^42^ Furthermore, sedating antihistamines have been found to reduce learning abilities in children.^44^ Notably, the risk of serious adverse drug effects increases with self-medication in children. For the treatment of cold and cough symptoms, parents often administer OTC medications to their child with insufficient knowledge about the medicine and potential risks of drug interactions.^45,46^ Further, prescription cold/flu medications used during the study period included antibiotics such as amoxicillin, azithromycin, and cefalexin. A growing body of evidence suggests that the gut microbiota plays a role in modulating the immune-system, and antibiotic treatment considerably alters the microbiome composition contributing to health complications.^6^ The findings of the current study suggest that EpiCor supplementation reduced the severity of cold/flu symptoms and therefore reliance on cold and flu medications, which may have detrimental effects in pediatric populations. Further, future studies could consider the differential effect of EpiCor on bacterial compared to viral infections as it relates to the use of cold/flu medications, including antibiotics.

In this study, there was no significant difference in the incidence of cold/flu symptoms between study groups, in contrast with previous research in adults. Previous randomized controlled trials conducted in adults over a 12-week study period demonstrated the beneficial effects of EpiCor postbiotic supplementation on cold/flu symptoms with reduced incidence^18,19^ and duration of cold/flu symptoms.^19^ Furthermore, adults supplemented with EpiCor had significantly fewer symptoms^19^ and a reduction in overall risk or incidence of cold/flu symptoms of more than 90% compared to placebo.^18^ While there is evidence of the beneficial effects of EpiCor supplementation in adults with a more developed immune system, further investigation of the effects of EpiCor in children aged 1-18 years was warranted. The safety and efficacy of EpiCor postbiotic supplementation in younger children was previously investigated in a randomized controlled trial (Mah, E. et al., 2019, Effects of a yeast fermentate formulated in a syrup with Vitamin C on cold or flu in generally healthy children: a randomized, double-blind, controlled, parallel trial, unpublished manuscript shared by the clinical trial sponsor with the authors of this study; clinicaltrials.gov identifier: NCT04093206). The study reported that EpiCor supplementation in children aged 1-6 years had a favorable safety profile but did not show significant differences in the maximum reported total CARIFS scores between IP and control groups, with ‘feels unwell’ as the only individual CARIFS item demonstrating a significant difference between groups. However, Mah et al. supplemented children with either a lower dose (7 mg/kg bodyweight) of EpiCor in combination with Vitamin C or Vitamin C alone as a comparator, marking notable differences in study methodology and design. The findings of Mah et al. led to the selection of the higher dose for the current study, which supplemented children with 500 mg of EpiCor or Placebo; neither supplement contained Vitamin C.

Further, age-related immune system differences may have played a role in eliciting the differing results in the two studies, as the current study enrolled an older age group of 4–12-year-olds. The immune system at birth is relatively immature and continues to evolve during childhood with exposures to different viral strains. A developing immune system in younger children makes them more susceptible to infections compared to those of an older age group. Immunity development in childhood occurs gradually because viral serotypes circulating within a community may not reappear for some years. Therefore, the immune system of the younger age group would be at a disadvantage compared to participants with a more developed immune system, as it may take years before a child acquires adequate protection during another encounter with the same viral serotype.

The mitigation of cold/flu severity may be just as important for children as lowering incidence. Influenza vaccines are inconsistently reliable in preventing disease, and providing adequate efficacy is associated with many challenges. The effectiveness of flu vaccine in mitigating and preventing influenza-associated illnesses and complications fluctuates each year. In the United States, the effectiveness of vaccination among 9–17-year-olds in 2021–2022 influenza season was 34% compared to only 7% in 2018-2019.^47,48^ Furthermore, differences in severity of viral strains from year to year add to the challenges of reducing the incidence of influenza from season to season.^49^ Nonetheless, even if a flu vaccine may not be able to prevent illness, clinicians and disease prevention and control programs across countries encourage vaccinations for the purpose of reducing severity of influenza-associated disease outcomes, including complications and mortality. Notably, countries with influenza vaccination programs have estimated reductions in severe disease and deaths.^50^ Although EpiCor supplementation did not result in a significantly lower incidence of cold/flu compared to Placebo, in line with vaccine research and medical recommendations, emphasis should be placed on demonstrated reductions in symptom severity.

This is the first clinical study in a pediatric population demonstrating efficacy of a postbiotic delivered in a gummy format. Nutraceuticals in a gummy format are especially important in children’s supplements because this population is less likely to tolerate more classical delivery forms such as capsules or tablets. Given the extreme temperatures and pH conditions used in the manufacturing process of gummies, demonstrating a nutraceutical’s efficacy in a gummy format is highly relevant. This study provides additional evidence supporting the safety and tolerability of a 500 mg dose of EpiCor in children. Among the 113 participants in the EpiCor group, there was a single case of diarrhea classified as a possibly related adverse event, which was moderate in severity and resolved three days after onset, representing 0.7% of the study participants. Furthermore, the US Food and Drug Administration, through two separate notifications, has assessed the safety of EpiCor. In dietary supplements, a 2011 New Dietary Ingredient 689 (NDI) notification determined a 500 mg dose to be safe for children aged 4 and older.^51^ For food use, 2020 Generally Recognized as Safe (GRAS) notice 928 confirmed the safety of EpiCor at 500 mg per serving, with a daily consumption limit of 3.8 g for ages 2 and older.^52^

This study did not include a follow-up period and therefore, the effect of EpiCor on cold/flu symptoms in the post-supplementation period could not be determined. Further, this study did not examine immunological blood markers such as blood lymphocyte populations, serum immunoglobulins, or cytokines. Future studies investigating mechanisms by which EpiCor exerts its effect on the developing immune system are warranted. Lastly, subgroup analyses based on vaccination status were not conducted in this study due to the small proportion of vaccinated children (<8%). Therefore, the efficacy of EpiCor in vaccinated vs. unvaccinated children is not known and should be explored. Given that the efficacy of a daily dose of 500 mg of EpiCor has now been demonstrated in children aged 4-12 years and in adults, future studies stratifying the study population into narrower age categories are warranted to better understand age-related differences in incidence and severity of cold/flu symptoms. Additionally, investigating the effects of 500 mg EpiCor supplementation in children outside of this population age group may provide a comprehensive understanding of how the developing immune system may influence the effect of EpiCor on the incidence, severity, and duration of cold/flu symptoms. Future studies should also focus on enrolling a more racially and ethnically diverse population compared to the current study. Minority children have been shown to experience a higher likelihood of flu-associated hospitalizations^53^ demonstrating the need to examine the efficacy of EpiCor in this population and generalizability of study findings.

## Conclusion

EpiCor supplementation resulted in significantly lower cold/flu severity compared to Placebo over the 84-day study period. These findings were further supported by EpiCor supplementation corresponding with a smaller proportion of participants utilizing cold/flu medication over the study period, thus reducing the possible risk of side effects. Importantly, supplementation with EpiCor was found to be safe and well tolerated in the studied population, strengthening previous safety findings. The combination of efficacy and safety findings from the current study suggest that EpiCor supplementation may allow caregivers to reduce the cold/flu medication provided to their child while also mitigating the severity of symptoms.

## Supplementary information

Supplementary Table

## Data Availability

The datasets generated during and/or analysed during the current study are available from the corresponding author upon reasonable request.
