# Supplementary material for: Efficacy of a yeast postbiotic on cold/flu symptoms in healthy children: A randomized-controlled trial
Source: Pediatr Res. 2024 Jun 28;96(7):1739–48. doi: 10.1038/s41390-024-03331-z (PMC11772231; doi:10.1038/s41390-024-03331-z)
Supplement: Supplementary file 1 — Supplementary Table [file 41390_2024_3331_MOESM1_ESM.docx]

**Efficacy of a yeast postbiotic on cold/flu symptoms in healthy children: a randomized-controlled trial**

**Authors:**

Ruma G. Singh^1^, Vicenta Garcia Campayo^2^, Justin Green^2^, Neil Paton^2^, Julissa D. Saunders^2^, Huda Al-Wahsh^1^, David C. Crowley^1^, Erin D. Lewis^1^, Malkanthi Evans^1,3^, Marc Moulin^1*^

**Author affiliations:**

^1^ KGK Science Inc., London, Ontario, Canada

^2^ Cargill Limited c/o Cargill Inc., Wayzata, Minnesota

^3^Department of Biochemistry, Western University, London, Ontario, Canada

***Corresponding author:**

Marc Moulin

KGK Science Inc., 275 Dundas Street, Tower A

Suite A1605, London, Ontario N6B 3L1, Canada

Email: [mmoulin@kgkscience.com](mailto:mmoulin@kgkscience.com)

Phone: (519) 438-9374

**Category of study:** Clinical Research Article

Supplementary Table 1. Specific antibiotic and inhaler use by participants during the study period

| **Group** | **Condition** | **Concomitant Medication Name** | **Days on Medication** |
| --- | --- | --- | --- |
| **Antibiotics** | | | |
| EpiCor | Pneumonia | Amoxicillin | 9 |
| EpiCor | Pneumonia | Amoxicillin | 10 |
| EpiCor | Pneumonia | Azithromycin | 4 |
| EpiCor | Pneumonia | Cephalexin | 9 |
| EpiCor | Infected molar | Amoxicillin | 9 |
| EpiCor | Cold | Amoxicillin | 8 |
| EpiCor | Ear infection | Clavulin | 6 |
| EpiCor | Ear infection | Ran-Cefprozil | 9 |
| EpiCor^a^ | Skin infection | Amoxicillin | 8 |
| EpiCor^a^ | Ear infection | Amoxicillin | 4 |
| EpiCor^a^ | Skin infection | Clindamycin | 4 |
| EpiCor | Ear infection | Amoxicillin | 6 |
| Placebo | Frequent throat infections | Amoxcillin | 7 |
| Placebo | Strep throat | Amoxicillin | 7 |
| Placebo | Ear infection and cough | Amoxicillin | 6 |
| Placebo | Sinus infection | Amoxicillin | 14 |
| Placebo | Ruptured ear drums bilaterally | Amoxicillin | 12 |
| Placebo | Tooth extraction | Amoxicillin | 7 |
| Placebo | Cold, cough and sore throat | Amoxicillin | 9 |
| Placebo | Ear infection and rash | Penicillin | 6 |
| **Inhalers** | | | |
| EpiCor^b^ | Cough and trachea tug/ indrawing | Ventolin | 1 |
| EpiCor^b^ | Cough and sore throat | Ventolin | 1 |
| Placebo^c^ | Sinusitis | Omnaris | 31 |
| Placebo^c^ | Runny and stuffy nose | Omnaris | 2 |
| Placebo | Congestion | Ventolin | Unknown^d^ |

^a, b, c^Multiple medications used by the same participant during the study period

^d^Participant continued use after the study period

Supplementary Table 2. CONSORT 2010 checklist of information to include when reporting a randomised trial

| Section/Topic | Item | Checklist item | Reported on page No |
| --- | --- | --- | --- |
| **Title and abstract** | 1a | Identification as a randomised trial in the title | 1 |
|  | 1b | Structured summary of trial design, methods, results, and conclusions (for specific guidance see CONSORT for abstracts) | 1 |
| Introduction - Background and objectives | 2a | Scientific background and explanation of rationale | 1-2 |
|  | 2b | Specific objectives or hypotheses | 2 |
| **Methods** |  |  |  |
| Trial design | 3a | Description of trial design (such as parallel, factorial) including allocation ratio | 2 |
|  | 3b | Important changes to methods after trial commencement (such as eligibility criteria), with reasons | N/A |
| Participants | 4a | Eligibility criteria for participants | 2 |
|  | 4b | Settings and locations where the data were collected | 2 |
| Interventions | 5 | The interventions for each group with sufficient details to allow replication, including how and when they were actually administered | 2 |
| Outcomes | 6a | Completely defined pre-specified primary and secondary outcome measures, including how and when they were assessed | 2 |
|  | 6b | Any changes to trial outcomes after the trial commenced, with reasons | N/A |
| Sample size | 7a | How sample size was determined | 3 |
|  | 7b | When applicable, explanation of any interim analyses and stopping guidelines | N/A |
| Randomisation: Sequence generation | 8a | Method used to generate the random allocation sequence | 2 |
|  | 8b | Type of randomisation; details of any restriction (such as blocking and block size) | 2 |
| Allocation concealment | 9 | Mechanism used to implement the random allocation sequence (such as sequentially numbered containers), describing any steps taken to conceal the sequence until interventions were assigned | 2 |
| Implementation | 10 | Who generated the random allocation sequence, who enrolled participants, and who assigned participants to interventions | 2 |
| Blinding | 11a | If done, who was blinded after assignment to interventions (for example, participants, care providers, those assessing outcomes) and how | 2 |
|  | 11b | If relevant, description of the similarity of interventions | 2 |
| Statistical methods | 12a | Statistical methods used to compare groups for primary and secondary outcomes | 3 |
|  | 12b | Methods for additional analyses, such as subgroup analyses and adjusted analyses | 3 |
| Results | | | |
| Participant flow (a diagram is strongly recommended) | 13a | For each group, the numbers of participants who were randomly assigned, received intended treatment, and were analysed for the primary outcome | 3 |
|  | 13b | For each group, losses and exclusions after randomisation, together with reasons | 3 |
| Recruitment | 14a | Dates defining the periods of recruitment and follow-up | 10 |
|  | 14b | Why the trial ended or was stopped | N/A |
| Baseline data | 15 | A table showing baseline demographic and clinical characteristics for each group | Table 1 |
| Numbers analysed | 16 | For each group, number of participants (denominator) included in each analysis and whether the analysis was by original assigned groups | 3 |
| Outcomes and estimation | 17a | For each primary and secondary outcome, results for each group, and the estimated effect size and its precision (such as 95% confidence interval) | 4-5 |
|  | 17b | For binary outcomes, presentation of both absolute and relative effect sizes is recommended | 4-5 |
| Ancillary analyses | 18 | Results of any other analyses performed, including subgroup analyses and adjusted analyses, distinguishing pre-specified from exploratory | 5 |
| Harms | 19 | All important harms or unintended effects in each group (for specific guidance see CONSORT for harms) | 5 |
| Discussion | | | |
| Limitations | 20 | Trial limitations, addressing sources of potential bias, imprecision, and, if relevant, multiplicity of analyses | 8 |
| Generalisability | 21 | Generalisability (external validity, applicability) of the trial findings | 8 |
| Interpretation | 22 | Interpretation consistent with results, balancing benefits and harms, and considering other relevant evidence | 6-8 |
| Other information | | |  |
| Registration | 23 | Registration number and name of trial registry | 10 |
| Protocol | 24 | Where the full trial protocol can be accessed, if available | 10 |
| Funding | 25 | Sources of funding and other support (such as supply of drugs), role of funders | 10 |

Citation: Schulz KF, Altman DG, Moher D, for the CONSORT Group. CONSORT 2010 Statement: updated guidelines for reporting parallel group randomised trials. BMC Medicine. 2010;8:18.
© 2010 Schulz et al. This is an Open Access article distributed under the terms of the Creative Commons Attribution License (<http://creativecommons.org/licenses/by/2.0>), which permits unrestricted use, distribution, and reproduction in any medium, provided the original work is properly cited.
